# Mining Disease Risk Patterns from Nationwide Clinical Databases for the Assessment of Early Rheumatoid Arthritis Risk

**DOI:** 10.1371/journal.pone.0122508

**Published:** 2015-04-13

**Authors:** Chu Yu Chin, Meng Yu Weng, Tzu Chieh Lin, Shyr Yuan Cheng, Yea Huei Kao Yang, Vincent S. Tseng

**Affiliations:** 1 Department of Computer Science and Information Engineering, National Cheng Kung University, Tainan, Taiwan; 2 Department of Internal Medicine, Division of Allergy, Immunology, and Rheumatology, National Cheng Kung University Medical College and Hospital, Tainan, Taiwan; 3 Health Outcome Research Center, National Cheng Kung University Hospital, Tainan, Taiwan; 4 Institute of Clinical Pharmacy and Pharmaceutical Sciences, National Cheng Kung University, Tainan, Taiwan; 5 Institute of Medical Informatics National Cheng Kung University, Tainan, Taiwan; 6 Telecommunication Laboratories, Chunghwa Telecom Co., Ltd., Taiwan; Irvine, UNITED STATES

## Abstract

Rheumatoid arthritis (RA) is a chronic autoimmune rheumatic disease that can cause painful swelling in the joint lining, morning stiffness, and joint deformation/destruction. These symptoms decrease both quality of life and life expectancy. However, if RA can be diagnosed in the early stages, it can be controlled with pharmacotherapy. Although many studies have examined the possibility of early assessment and diagnosis, few have considered the relationship between significant risk factors and the early assessment of RA. In this paper, we present a novel framework for early RA assessment that utilizes data preprocessing, risk pattern mining, validation, and analysis. Under our proposed framework, two risk patterns can be discovered. Type I refers to well-known risk patterns that have been identified by existing studies, whereas Type II denotes unknown relationship risk patterns that have rarely or never been reported in the literature. These Type II patterns are very valuable in supporting novel hypotheses in clinical trials of RA, and constitute the main contribution of this work. To ensure the robustness of our experimental evaluation, we use a nationwide clinical database containing information on 1,314 RA-diagnosed patients over a 12-year follow-up period (1997–2008) and 965,279 non-RA patients. Our proposed framework is employed on this large-scale population-based dataset, and is shown to effectively discover rich RA risk patterns. These patterns may assist physicians in patient assessment, and enhance opportunities for early detection of RA. The proposed framework is broadly applicable to the mining of risk patterns for major disease assessments. This enables the identification of early risk patterns that are significantly associated with a target disease.

## Introduction

Rheumatoid arthritis (RA) is a chronic autoimmune rheumatic disease most commonly occurring in older patients and females. Severe symptoms include painful swelling in the lining of the joints, morning stiffness, and joint deformation and destruction. The prevalence of RA is 1% in the global population [1] and 0.09% in Taiwan [2]. The pathogenesis of RA is unknown; there are still many interpretations of the disease, and research in this area is ongoing. RA can be easily controlled in its early stages with pharmacotherapy, but diagnosis at this point is difficult. RA is typically diagnosed when the patient is seriously ill with severe symptoms, at which point the disease is beyond effective treatment. If not treated early, RA patients suffer persistent and permanent bone and joint destruction, decreased quality of life, and even reduced life expectancy.

To improve treatment quality, medical organizations have accumulated vast amounts of clinical information. The effective use of this information in medical decision-making requires analysis software to mine the considerable expertise. Many data mining techniques use treatment decisions for disease assessment models; moreover, related system applications and their algorithms are designed based on differing medical data characteristics.

Recent work on data mining in medical applications has involved hepatitis B surface antigen (HBsAg) immunoassay prediction [3], survival rate prediction [4], [5], prescription analysis [6], and comorbidity analysis [7]. These studies applied several data mining techniques, including associative rule mining [8], support vector machines [9], C4.5 decision trees [10], and neural networks [11]. Medical informatics and decision support systems are effective applications for addressing problems with diverse features and various data categories, thereby fostering the development of these algorithms.

Currently, the diagnosis of RA involves an evaluation of the patient based on clinical experience using certain RA disease classification criteria (such as the 1987 and 2010 American College of Rheumatology (ACR)/European League Against Rheumatism (EULAR) Classification Criteria for Rheumatoid Arthritis [12]). If early RA symptoms are accurately assessed, and the appropriate treatment is promptly administered, patients can avoid permanent damage to the normal health and development of their bones and joints, improving their quality of life.

Various methods for the early diagnosis of RA have been proposed, such as the aforementioned 1987 and 2010 ACR/EULAR Classification Criteria [12], the van der Helm–van Mil (vHvM) score [13], and the antibodies against cyclic citrullinated peptide (anti-CCP) prediction factor [14]. In existing research on disease assessment, records of patients’ persistent symptoms are extremely valuable. However, they are difficult to integrate into electronic medical records because of the long-term accumulation of patient information across different institutions, divisions, and locations. Previous studies have tended to collect the RA patient cohort from specific regional hospitals, rather than on a nationwide basis. Moreover, to the best of our knowledge, data mining techniques have never been applied to RA disease assessment or the decision support of RA diagnoses.

For the present retrospective cohort study, we designed a novel framework that enables the analysis of a vast nationwide clinical database. To support RA disease assessment and analysis, our approach mines the class association rules (CARs) of RA before making a definite diagnosis. The effectiveness of the patterns obtained in this way is evaluated by a *k*-fold validation method that uses measured sensitivity and specificity values. The risk patterns can assist physicians in patient assessment, and enhance opportunities for the early detection of RA. Consequently, it is possible to provide early treatment to RA patients, reducing damage to bones and joints, and improving patient quality of life.

## Related Work

In this disease assessment analysis, we investigate the factors involved in RA disease prediction, data mining applications in the prediction of other diseases, data mining of the Taiwan National Health Insurance Research Database (NHIRD), and associated classification techniques.

### Rheumatoid Arthritis Prediction

The prediction of RA is highly feasible. Previous studies have considered various physiological attributes and statistical regression to establish disease prediction models. For instance, Shadick et al. [15] used C-reactive protein to predict the likelihood of RA, whereas Huizinga et al. [16] examined nine clinical variables (gender, age, localization of symptoms, morning stiffness, tender and swollen joint count, C-reactive protein, rheumatoid factor, and anti-CCP antibodies) to construct prediction rules. Cader et al. [12] used the 2010 and 1987 ACR/EULAR criteria for the prediction of RA patients from a very early synovitis cohort, and Bedran et al. [14] employed a new patient cohort to validate the vHvM score in the prediction of RA in patients with recent onset of undifferentiated arthritis.

### Data Mining in Disease Prediction

Currently, many data mining techniques are used in disease prediction to address related problems. In recent years, the data mining of electronic medical records has become quite popular, and further results are highly anticipated. The use of data mining is expected to decrease costs and the public health burden, and should improve diagnostic accuracy, prevention, treatment, and quality of care. Mathias et al. [4] used predictive data mining and high-dimensional analytics of electronic health record data from elderly patients to develop a highly accurate and clinically actionable five-year life expectancy index. Shang et al. [3] used a decision tree technique and logistic regression with blood testing data, including alanine aminotransferase, serum albumin, and alkaline phosphatase, to predict the HBsAg immunoassay. Their method replaces immunoassays or assays not readily available for a laboratory diagnosis of hepatitis B infection. Ng et al. [5] developed a decision support tool to predict the survival of cancer patients beyond 120 days after palliative chemotherapy. With further validation, this tool, coupled with the professional judgment of clinicians, can help improve patient care.

### Data Mining with the Taiwan NHIRD

The objective of data mining is to extract important information and reveal implicit patterns. If source data is not readily available, and the data size is very large, there are numerous opportunities for mining with appropriate rules. The Taiwan NHIRD, a nationwide clinical database, is such a case. In recent years, a growing number of studies have applied data mining techniques to the NHIRD. This research includes an analysis of comorbidities (other diseases occurring with the primary disease) and analysis of specific disease treatment drugs (associations among drugs). Chen et al. [6] used association rule mining techniques to analyze Chinese herbal medicine prescriptions for asthmatic children using the NHIRD, and Tai [7] applied association rule mining techniques to explore the complex network of ADHD comorbidity and the practicality of association rule mining in comorbidity studies using the NHIRD.

### Associative Classification Method

Associative classification integrates features of association rule mining and classification rule mining. The function of classification rule mining is to discover a small set of rules that forms an accurate classifier. Association rule mining identifies all rules in the database that satisfy specific minimum support and minimum confidence constraints [17].

Classification based on multiple association rules (CMAR) [18] is an associative method. It extends the efficient frequent pattern mining method, which is much faster than a priori-like methods, to achieve a better accuracy rate, and is suitable for mining large databases. In addition, it efficiently stores and retrieves mined class-association rules, and effectively prunes rules based on confidence, correlation, and database coverage. The classification is performed based on weighted analysis using multiple strong association rules. In our approach, we apply this method to the RA assessment framework to identify rules of symptoms/diseases with a high correlation to RA. These rules are then integrated to assess patient RA risk before making a definite diagnosis.

## Methods

### Framework and Workflow

**Fig 1:**
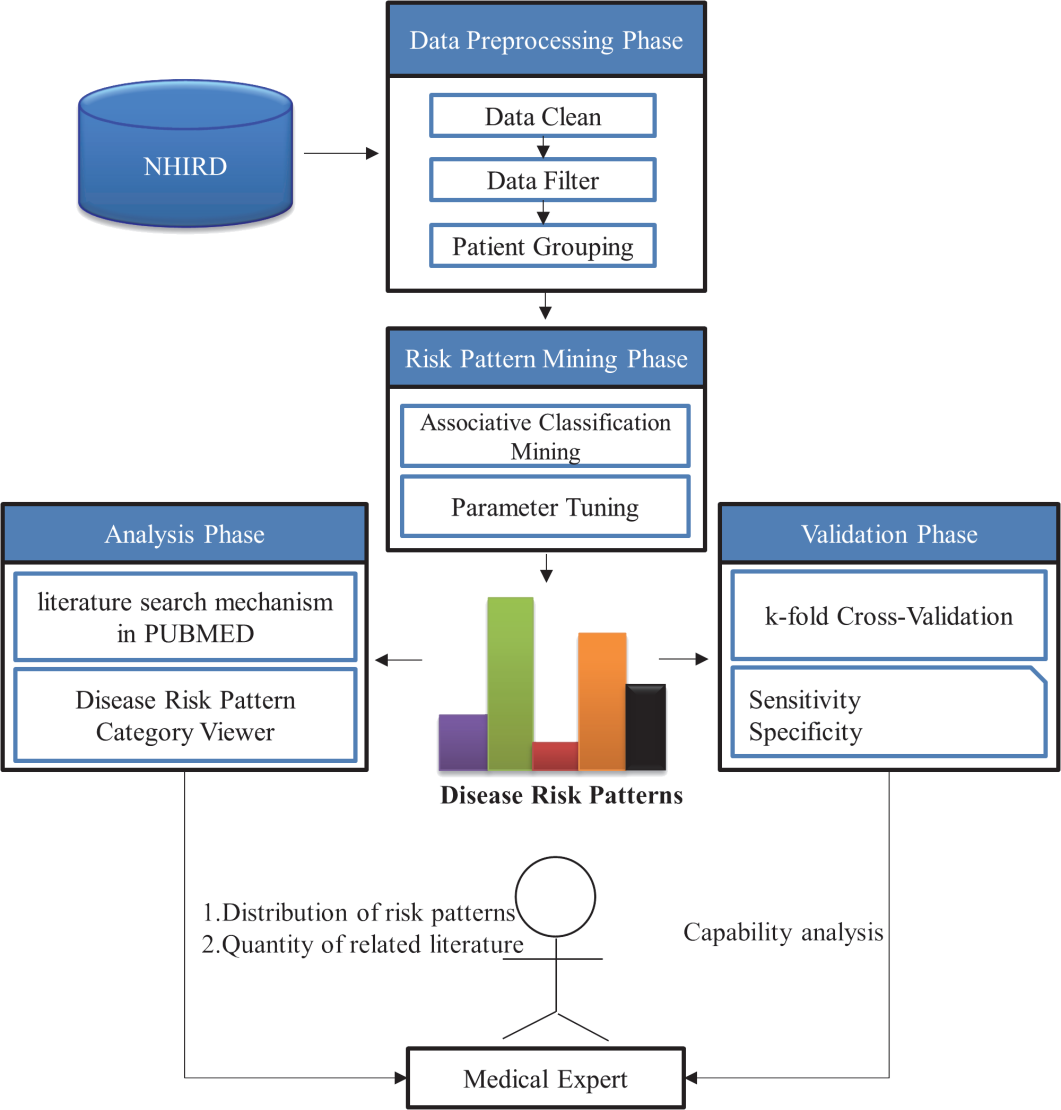
Workflow of data mining process for RA disease early detection.

In this study, we describe a novel framework for the mining and analysis of RA risk patterns with the aim of facilitating the early detection of RA disease. The framework has four phases: data preprocessing, risk pattern mining, risk pattern validation, and analysis (see Fig. 1). In the preprocessing phase, the data extracted from the NHIRD is analyzed. These data include patient IDs, outpatient dates, and diagnosis coding from the International Classification of Diseases, Ninth Revision, Clinical Modification (ICD-9-CM). Data input errors are then excluded. According to RA diagnostic criteria, data is divided into RA disease groups and a non-RA disease group. In the data mining and validation phases, we employ a ten-fold validation method using training and test datasets. Associative classification mining is then applied to discover general RA disease risk patterns with which to form the risk model. We evaluate the capability of the RA disease risk models by evaluating their sensitivity and specificity.

In the analysis phase, we use the National Center for Biotechnology Information (NCBI) E-utilities Web Service (SOAP) to design an evaluation and automation mechanism for risk pattern-related literature. The inferential RA disease risk patterns that we identify are novel and build a new, potentially strong hypothesis, a point based on the small amount of related literature. Finally, to provide further characterization information and pattern distributions for comparative analysis by medical experts, we describe the Risk Pattern Category Viewer, a tool that represents and organizes risk patterns based on a tree structure. In the following, we describe the details of the proposed framework.

### Data Preprocessing Phase

#### Data Cleaning

Data in which coding errors may occur for the ID, sex, date of birth, or diagnosis code fields are removed.

#### Filtering

Patient data with fewer than four diagnostic records are filtered. If the number of diagnostic data records in a patient’s medical history is too small, it is essentially meaningless, as there is little recognition or representation. Hence, such patients are removed from the cohort.

#### Patient Grouping

Patient grouping is based on the cohort-extracted clinical data of patients from the NHIRD who were confirmed as having an RA diagnosis and catastrophic illness record. The patient data are divided into an RA group and a non-RA group. The RA patient definition is provided below.

#### RA Patient Definition

**Fig 2:**
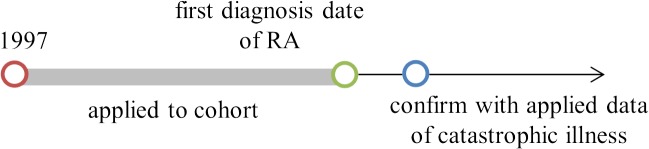
Timeline for data collection of RA group.

For our purposes, the definition of an RA patient is one whose first RA diagnosis was between 1997 and 2008, and whose RA catastrophic illness was confirmed in the registry record with ICD code 714.0 and A-code 430 (denoting RA in clinical and registry records). In the NHIRD, the A-code was used before December 31, 1999, and the ICD-9-CM code was used during the period 2000–2008. The cohort-collected clinical records are from before the first diagnosis of RA for each patient. The timeline for data collection of the RA group is shown in Fig. 2.

#### Non-RA Patient Definition

Non-RA patients are those without RA; that is, a patient from the cohort with no catastrophic illness record of RA diagnosis during the period 1997–2008. These non-RA patients comprise the non-RA group.

### Risk Pattern Mining Phase

#### Associative Classification by CMAR [18]

The proposed framework integrates efficient associative classifiers to mine RA risk patterns from a large, nationwide clinical database. The risk patterns are rule-based, easy to understand, and contribute to clinical decision support with disease assessment. In this phase, we extract risk patterns that occur frequently, and classify the disease status of the patient. A given database of NHIRD diagnosis records is denoted as transaction ***T***. Let object ***obj*** = (D1, …, Dn), where D1, …, Dn are the patient diagnosis records. The diagnosis adheres to ICD-9-CM. Pattern ***P*** = D1, …, Dn is a set of disease codes that occur in the same patient. Let ***c*** be class labels that indicate an RA group or non-RA group.

For rule ***R***: ***P*** ➔ ***c***, the number of data objects in ***T*** matching pattern. These have the class label ***c***, which is called the ***support*** of ***R*** and is denoted as ***sup***(***R***). The ratio of the number of objects of matching pattern ***P*** that have class label ***c*** versus the total number of objects with matching pattern ***P*** is called the **confidence** of *R*, denoted as ***conf***(***R***). For example, if 91% of patients have been diagnosed with Sjogren’s syndrome before a definite diagnosis of RA—i.e., the confidence of rule ***R***: ***Sjogren’s syndrome*** ➔ ***RA*** is 91%—then we can use many rules ***R*** to assess the patient clinical data. When a new (unknown) patient enters the cohort, the classifier selects the rule that matches the new patient clinical data and has the highest confidence with support. This information is used to predict the class label of the new patient.

To prevent important rules from being ignored—i.e., those that are slightly low in confidence but high in support—CMAR proposes a weighted *x* ^*2*^ to integrate correlation and popularity information into the measure. This improves the objectivity of the results. In our experiments, we tested different parameter adjustments, and set a suitable threshold for the support and confidence so that meaningless rules would be filtered. We also allowed CMAR to locate the complete set of CARs, and enhanced the sensitivity and specificity of the models.

### Validation Phase

#### K-fold Cross-validation

For validation purposes, we applied ten-fold cross-validation. The cohort was randomly split into ten subsamples: one formed the test dataset for verifying the effectiveness of the model, and the others formed the training dataset to determine RA disease risk patterns for the model. The cross-validation process was repeated ten times, with each of the ten subsamples used as the validation data once.

To evaluate the models and analyze their assessment capabilities, we measured the sensitivity and specificity of each model. The sensitivity of a model is its ability to correctly classify an individual as “diseased.” We evaluate this metric as$\frac{\sum True\;positive}{\sum Condition\;positive}$, where a true positive is an RA patient who is correctly predicted as having RA. Specificity is the ability of a test to correctly classify an individual as “non-diseased.” The equation for this metric is$\frac{\sum True\;negative}{\sum Condition\;negative}$, where a true negative is a non-RA patient correctly predicted as not having RA.

### Analysis Phase

#### Well-known-degree Analysis of Patterns

The NHIRD mining framework can be used to mine interesting and effective patterns if the partial of discovered patterns are well known and consistent with known medical knowledge. This finding can be new significant discoveries or supports related literature based on population, particularly if the patterns have not yet been reported or have been less frequently reported. In this case, they meet a minimum support value with a high confidence value, and enhance the accuracy of the RA assessment.

Therefore, to analyze the novelty of risk patterns in previous studies, we applied SOAP [19], which provides a stable interface for accessing the PubMed literature database. Our aim is to design an automated mechanism for calculating the quantity of pattern-related works available in PubMed. PubMed comprises more than 23 million citations for biomedical literature from MEDLINE, life science journals, and books. We used the following search pattern: *disease 1 of pattern* ***[TIAB] AND*** *disease 2 of pattern* ***[TIAB] AND*** *disease 3 of pattern* ***[TIAB]*** …, until a specific pattern was matched. “[TIAB]” refers to the literature title, collection title, abstract, and other excerpts of a citation.

#### Risk Pattern Viewer

**Fig 3:**
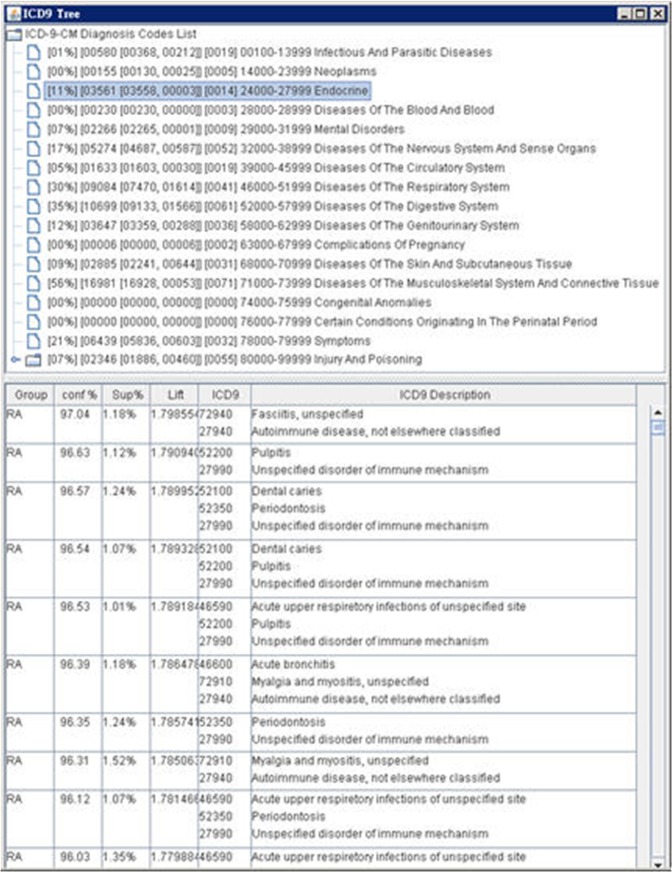
Risk Pattern Viewer.

We designed a tree-structure-based Risk Pattern Viewer to enable physicians to further analyze risk pattern characteristics and distributions. The user interface is split into two parts (see Fig. 3), which are described as follows:

Part I: The Patterns Distribution View shows a list with segments assigned by the ICD-9-CM coding system. ICD-9-CM has different segments for various disease classifications. The interface aligns with these segments, and provides relevant information:

1. Field 1: Ratio of all related patterns in this ICD-9-CM coding segment.
2. Field 2: Distribution of patterns among all patients, the RA group, and the non-RA group in the specific ICD-9-CM coding region.
3. Field 3: Number of patterns of unique diseases in the RA group in the specific ICD-9-CM coding region.
4. Field 4: Specific ICD-9-CM coding region.
5. Field 5: Description of the specific ICD-9-CM coding region.

Part II: The Pattern Properties View is employed when a user selects a specific field of the ICD-9-CM coding region in the Patterns Distribution View. The Pattern Properties View shows the pattern characteristics, which can be sorted by the following attributes:

1. Group: The group to which the pattern applies.
2. Confidence (Conf.): Confidence is defined as the probability of seeing the risk pattern’s consequences under the condition that the medical transactions also contain the antecedent [8]. The formula for the confidence of a risk pattern is:$conf(X\Rightarrow Y)=\frac{\text{supp}(X\cup Y)}{\text{supp}(X)}=\frac{P(X\begin{array}{c} and \end{array}Y)}{P(X)}=P(Y|X)$; Let X represent related-diseases or symptoms that are high-risk factors implying RA or non-RA, and let Y represent RA or non-RA as a target disease. For example, the risk pattern {Eczema, asthma, urinary tract infections➔ RA} has a high confidence of 86.55%, which represents that a patient diagnosed with the disease/symptom items of eczema, asthma, and urinary tract infections has a high risk of being diagnosed with RA.
3. Sup. %: Support supp(X) of item set X, defined as the proportion of transactions in the specific group that contains the item set.
4. Lift (originally called interest): The lift of a risk pattern is defined as $lift(X\Rightarrow Y)=\frac{conf(X\Rightarrow Y)}{\text{supp}(Y)}=\frac{\text{P}(XandY)}{\text{P}(X)\times \text{P}(Y)}=\frac{\text{supp}(X\cup Y)}{\text{supp}(X)\times \text{supp}(Y)}$. Lift is used to measure how many times more often diseases X and Y (target disease) occur together than would be expected if they were statistically independent [20]. If the lift value is greater than 1, the occurrence of the each disease is dependent on the other. Thus, the risk pattern is potentially useful for risk prediction.
5. ICD-9-CM and the description field are the ICD-9-CM code region and disease or symptom description, respectively.

## Materials

The cohorts were gathered from Taiwan NHIRD clinical records, provided by the Taiwan National Health Insurance (NHI) Administration, Ministry of Health and Welfare. The NHI coverage rate at the end of 2007 was 99.3% [2], almost the total population of Taiwan, which thereby makes the NHIRD a nationwide database. The national representation of these data enables analysis across different hospitals, divisions, and regions. The data we consider include a 12-year follow-up period for a million patients. Previous studies on patient dataset characteristics for RA risk patterns considered only an 18-month follow-up for a few hundred patients [20].

NHIRD data accumulated over many years is suitable for longitudinal studies. In this study, we collect 12 years of clinical records from 1997 to 2008. Based on these records, we aim to discover the diseases that are highly correlated with RA, and thus form risk patterns for assessing the risk of RA.

The NHIRD clinical record code format follows ICD-9-CM. This is used to represent clinical diagnosis records; a maximum of three clinical diagnosis records can be assigned on each visit.

### Statistical Analysis

The frequency and distribution of continuous variables for all patients were compared using the Student’s t-test. The results are given in Table 1. The significance test was based on a standard deviation derived from the standard deviations of the RA group and non-RA group. The threshold for alpha (Type I) error, although generally accepted as 0.05, was found to be rather sensitive to sample size.

**Table 1:** Clinical course variables among patients categorized by RA diagnosis and gender.

Characteristic	Sex	RA group	Non-RA group	p-value
**Age, mean ±SD**	All	58.7±15.4	42.5±20.6	0.00
	Female	58.1±14.9	42.53±20.5	0.00
	Male	60.5±16.8	42.4±20.7	0.00
**Number of patients**	Female	1,013	470,520	0.00
	Male	301	494,759	0.00
**Data source**	F:485,934 M:513,703			
**Year**	1997–2008			
**Source**	National Health Insurance Research Database (NHIRD)			

## Experiments

The cohorts of our study included an RA group and a non-RA group, as previously defined. The RA group of 1,314 patients included 1,013 women and 301 men. Clinical records from the 12-year period 1997–2008 were gathered. For a group comparison, we also obtained non-RA patient clinical data of 965,279 patients from 1997–2008.

A comparative analysis between the RA group and non-RA group showed significant differences in the age and number of patients. The average age of the RA group was greater than that of the non-RA group. There were significant differences, with a p-value of 0.00. The clinical course variables among the patients, categorized by RA diagnosis and gender, are listed in Table 1.

**Fig 4:**
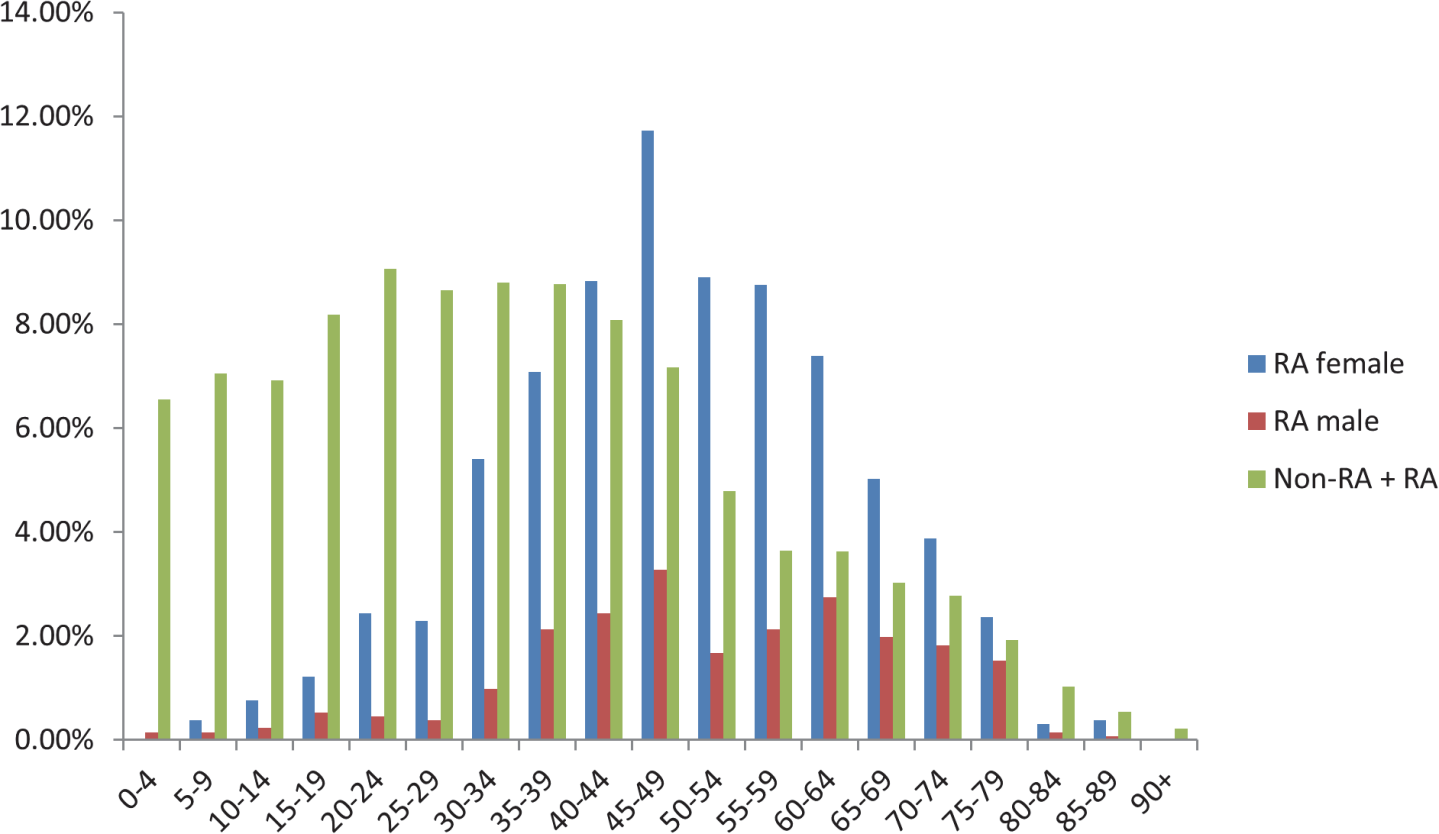
Age distribution of cohort.

The age interval containing the maximum number of patients was the 45–49 years range, and 63.14% of female patients were in the 30–70 years range. The data indicate that the age of RA patients followed a normal distribution. The age distribution of the cohort is shown in Fig. 4.

### Disease Risk Pattern Evaluations under Different Parameter Settings

#### Support and Confidence Thresholds

**Fig 5:**
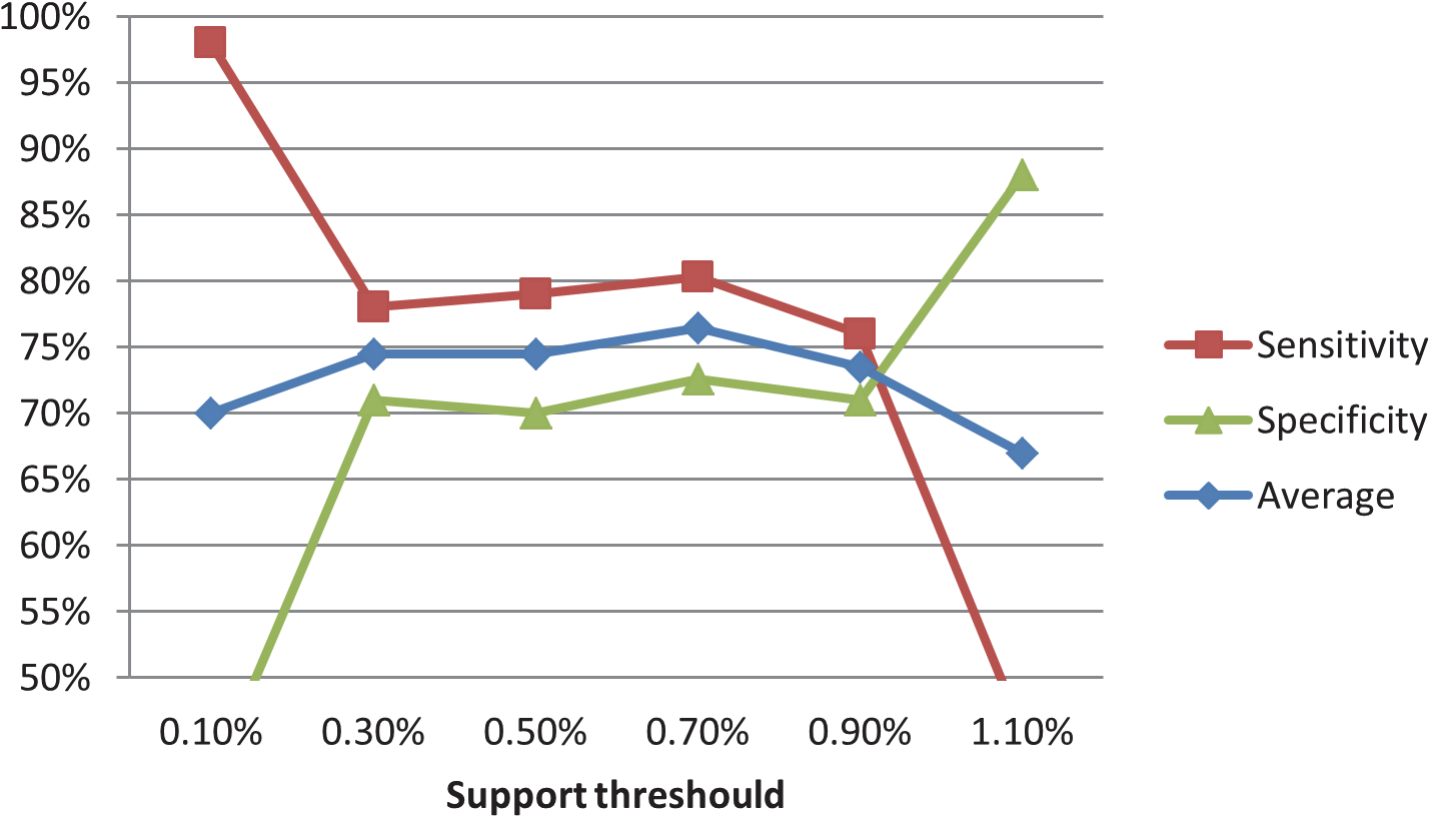
Trend of RA assessment effects under different support thresholds.

We set the minimum confidence threshold value to 50%. That is, patterns with a confidence value of greater than 50% are assumed to have the ability to classify the disease [17], [18], [21]. The support threshold varied according to the disease characteristics, pattern characteristics, and disease prevalence. To determine a suitable support threshold, we experimented with support thresholds ranging from 0.1% to 1.1% at intervals of 0.2%. The most suitable value was identified to be 0.7% by considering the optimal average of sensitivity and specificity, as well as the trend of RA assessment effects (see Fig. 5).

#### Omission of Unrepresentative Diagnostic Records

**Fig 6:**
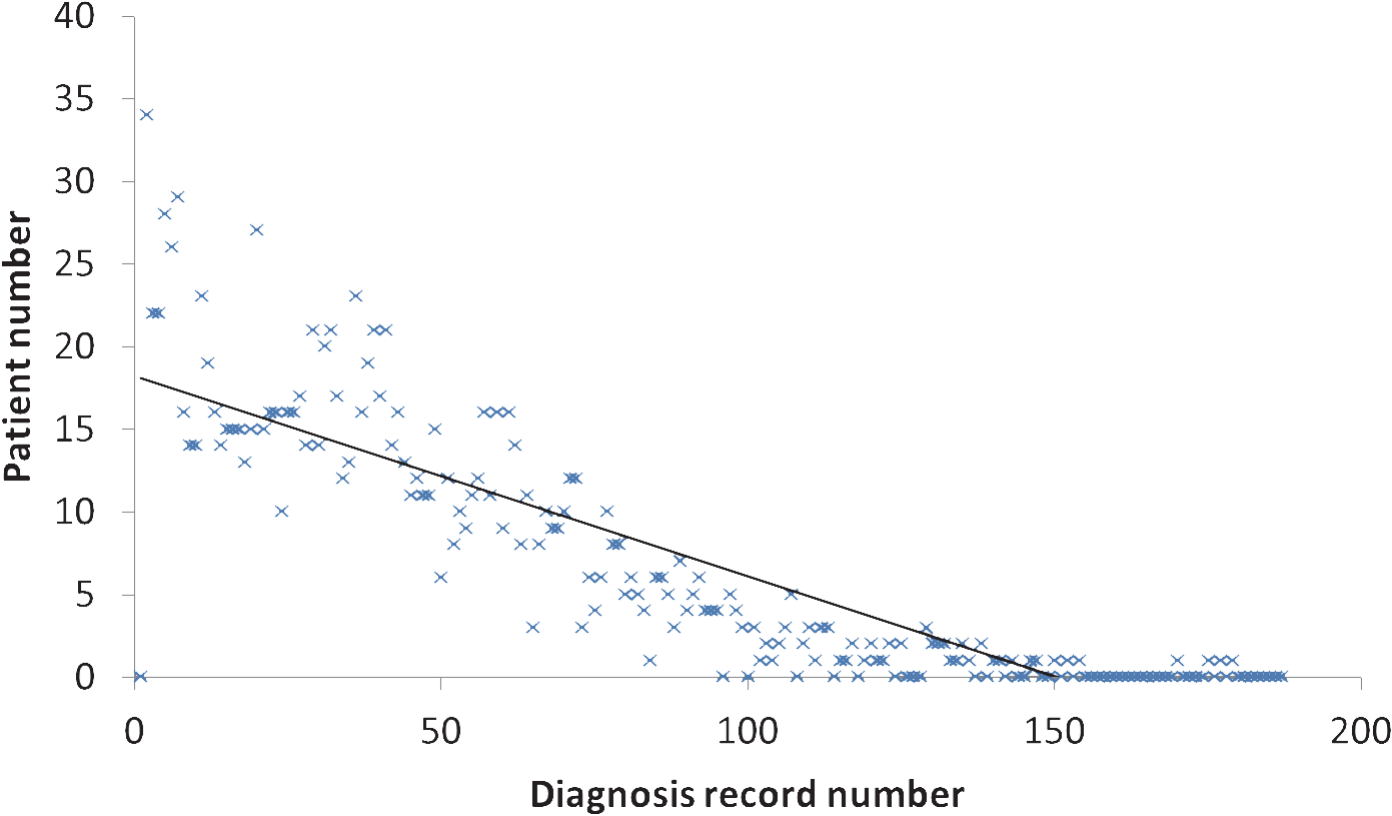
Distribution of RA patients under different numbers of diagnosis records.

A small number of patient diagnosis records were unrepresentative and required removal. Patients who lacked a sufficient number of diagnosis records provided insufficient information to implicate a disease risk model, and could therefore not provide implicit information for early disease assessment analysis. The distribution of RA patients under different numbers of diagnosis records is illustrated in Fig. 6. As shown in Fig. 6, such partial patient diagnosis records are rare. However, if we removed data concerning patients with sufficient diagnosis records to imply a disease risk model, we would have mistakenly deleted meaningful information.

**Fig 7:**
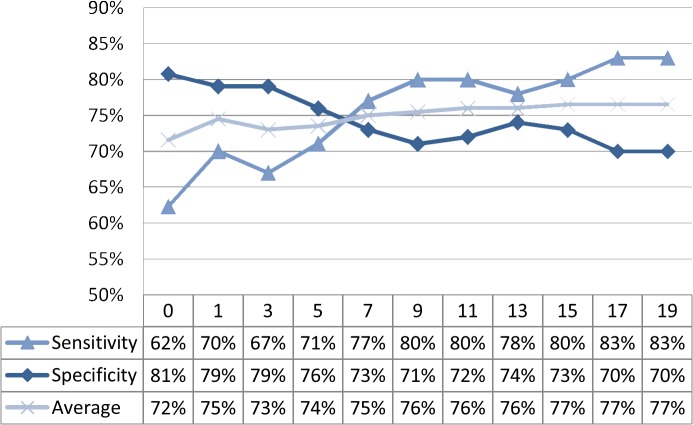
Trend of RA assessment effects under different diagnosis record numbers.

Following discussions with physicians and experiments with different diagnosis record filter values, we determined that the removal of patients with fewer than nine diagnostic records was appropriate; this number is approximately equivalent to three visits to a doctor. Therefore, to maintain the validity of the experiments, the minimum number of diagnosis records was set to 15. The trend of RA assessment effects under different diagnosis record filter numbers is shown in Fig. 7.

## Results and Discussion

### Mining Significant Disease Risk Patterns

In our study, the format of disease risk patterns was {***disease diagnosis*** ➔ ***RA or non-RA*, *confidence value*, *support value*, *relative risk value}***, where “➔” denotes implication. The relative risk value is the risk ratio of the pattern occurring in the implied group compared to another group.

To analyze these important risk patterns in terms of combinations of diseases or symptoms, we screened disease types from the risk pattern set. The number of disease occurrences of the RA group was 3,169 before mining the 250 million NHIRD outpatient records. After mining, we had 32,560 RA risk patterns. From Table 2, we can see there are 497 kinds of diseases and symptoms that appear in RA risk patterns, accounting for about 16% of the total. That is, the mining framework can filter most of the non-related diseases, and thus mining of significant disease risk patterns can assist physicians in focusing their analysis on the frequent occurrences of RA for patient premorbidity. Hence, only those diseases with a strong correlation will be applied to clinical trials. The characteristics of the discovered patterns are given in Table 2.

**Table 2:** Characteristics of mined patterns.

	RA group	Non-RA group
**Number of clinical records**	281,208	227,453,049
**Number of diseases**	3,169	16,518
**Number of risk patterns**	32,560	2,914
**Number of diseases that occur in disease risk patterns**	497 (16%)	308 (2%)

### Distribution of Disease Risk Patterns in the ICD-9-CM Coding System

The distribution of disease risk patterns according to the ICD-9-CM coding system is summarized in Table 3. We calculated the relative risk of the disease risk patterns between the RA group and the non-RA group. This analysis enables physicians to understand which patterns display significant differences in the respective disease categories.

**Table 3:** Risk pattern distribution between the RA group and non-RA group for all diseases categorized in ICD-9-CM.

Disease Category	ICD-9-CM code	RA	Non-RA	RR	CI (95%)
**Infectious and parasitic**	001–139	2.46%	7.62%	0.32	(0.27–0.37)
**Neoplasm**	140–239	1.92%	0.31%	6.21	(5.71–7.28)
**Endocrine, metabolic, and immunity**	240–279	9.70%	0.55%	**17.66**	(15.36–21.74)
**Blood and blood-forming organs**	280–289	1.66%	0.03%	**48.24**	(29.23–286.57)
**Psychiatric disorders**	290–319	9.85%	0.14%	**71.78**	(51.16–144.86)
**Nervous system and sense organs**	320–389	20.10%	18.74%	1.07	(1.04–1.10)
**Circulatory system**	390–459	10.54%	0.82%	**12.8**	(11.55–14.79)
**Respiratory system**	460–519	31.45%	44.89%	0.7	(0.68–0.72)
**Digestive system**	520–579	35.32%	42.62%	0.83	(0.81–0.85)
**Genitourinary system**	580–629	16.06%	6.49%	2.48	(2.46–2.49)
**Complications of pregnancy, childbirth, and puerperium**	630–679	0%	0.58%	0	(0.00–0.00)
**Diseases of the skin and subcutaneous tissue**	680–709	8.52%	18.98%	0.45	(0.41–0.48)
**Musculoskeletal system**	710–739	57.18%	1.30%	**43.85**	(38.33–51.8)
**Congenital anomalies**	740–759	0%	0%	0	(0.00–0.00)
**Conditions prenatal period**	760–779	0%	0%	0	(0.00–0.00)
**Symptoms, signs**	780–799	21.91%	16.61%	1.32	(1.29–1.35)
**Injury and poisoning**	800–999	9.52%	17.02%	0.56	(0.52–0.60)

To compare the different distribution rates of disease risk patterns in the RA group and non-RA group, we applied a relative risk ratio analysis. The ratio was calculated by dividing the disease risk pattern rate of the RA group by that of the non-RA group in the same ICD-9-CM categories. The significance test was based on a new standard deviation derived from the standard deviations of the RA group and non-RA group. The threshold of alpha (Type I) error, although generally accepted as 0.05, was found to be rather sensitive to the sample size. This analysis demonstrates the potential for RA early detection through segments that strongly differentiate the RA group from the non-RA group.

The risk pattern distribution of the RA group and non-RA group for all diseases categorized in ICD-9-CM is given in Table 3. The distribution is ordered by the ICD-9-CM code value. The five most frequently occurring relative risks for the disease risk patterns are 290–319 psychiatric disorders [71.78, 95% Confidence interval (CI): (51.16–144.86)], 280–289 blood and blood-forming organs [48.24, 95% CI: (29.23–286.57)], 710–739 musculoskeletal system [43.85, 95% CI: (38.33–51.8)), 240–279 endocrine, metabolic, and immunity [17.66, 95% CI: (15.36–21.74)], and 390–459 circulatory system [12.8, 95% CI: (11.55–14.79)]. In addition, the musculoskeletal system category had a high relative risk value, and a very high risk pattern, in the RA group (57.18%).

#### Literature search with risk-related disease extracted from disease risk patterns

We searched related literature in the PubMed database to analyze which disease risk patterns were known to relate to RA. Our objective was to comprehend the novelty of the patterns and the degree to which they were known.

**Fig 8:**
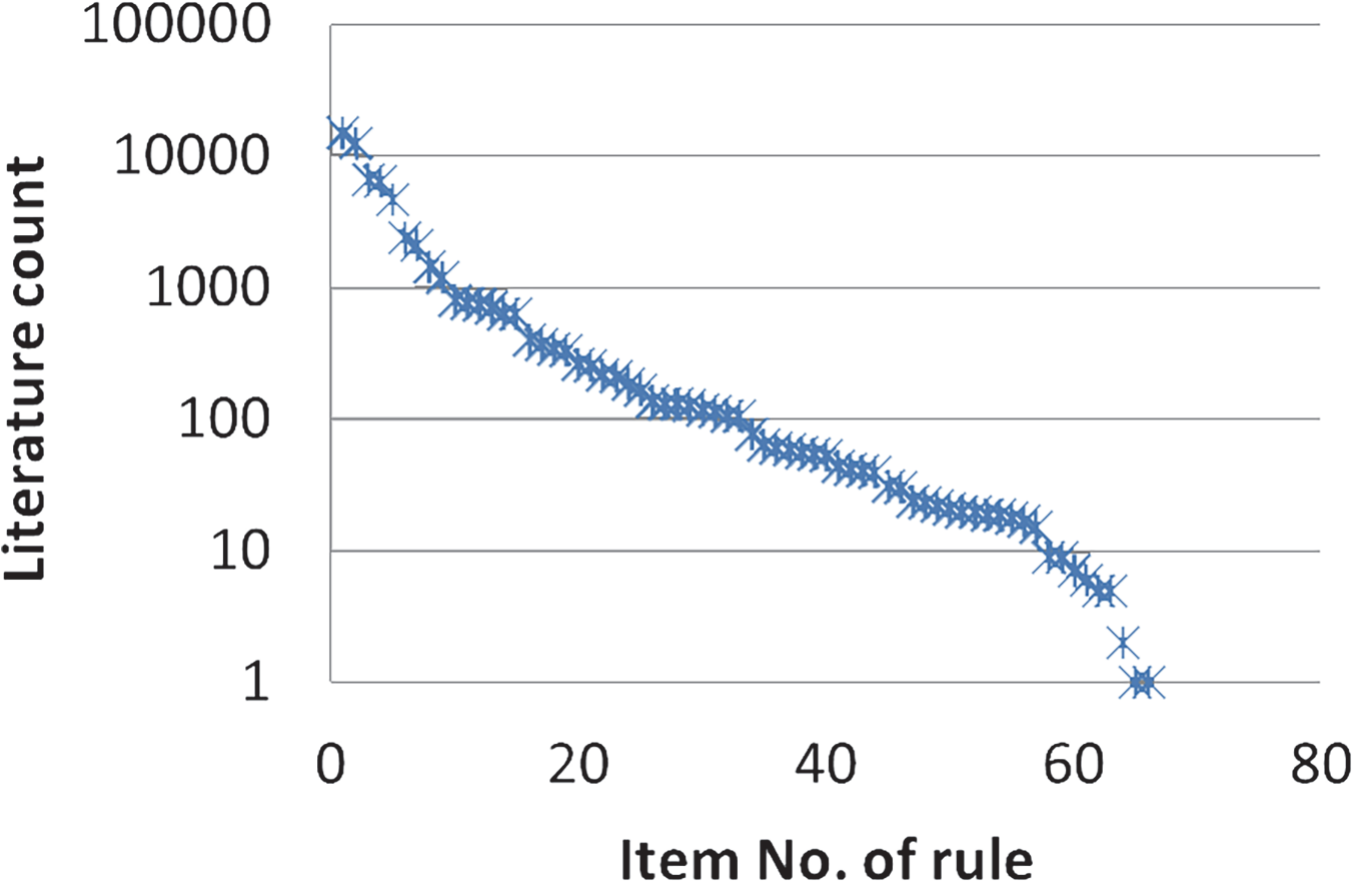
Trend in literature for single disease risk patterns in PubMed.

The format of the risk patterns was ***related***_***disease*** ➔ ***RA***, with the query term ***related_disease[TIAB] AND Rheumatoid arthritis[TIAB]***. The related disease name was a specific disease word extracted from the risk pattern, such as Meniere’s disease. The literature in PubMed exhibits an exponential trend for single diseases, as shown in Fig. 8. This trend demonstrates that the majority of single diseases in the risk patterns discovered by our framework are consistent with the existing literature. Only a small number of single diseases were not reported; that is, their association with RA is unconfirmed. Nevertheless, they have the potential to be included as novel findings.

#### Literature Search with Risk Patterns

**Fig 9:**
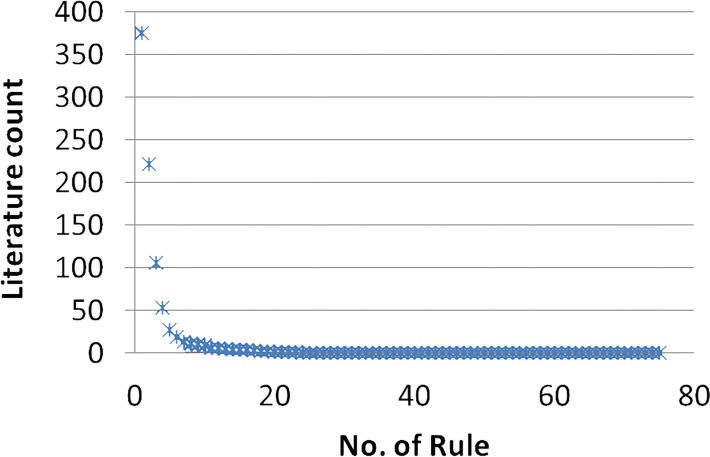
Trend in literature for PubMed pattern related mining.

As depicted in Fig. 9, the majority of disease risk patterns comprising two or more diseases have not been reported in the literature. However, a small number of patterns were reported that consisted of only a single disease.

As shown by Figs. 8 and 9, most past research has studied the relationship between a single disease and RA; however, the relationship between multiple diseases and RA has been addressed less often. For example, mitral valve disorder and RA was covered in 41 studies, and conjunctivitis and RA was covered 25 times. However, no papers discuss RA, mitral valve disorder, and conjunctivitis together. The disease risk pattern of **mitral valve AND chronic conjunctivitis** ➔ **RA** showed a confidence level of 83.57% with high lift dependencies, which indicates that these two diseases have occurred in the same patient before a definite diagnosis of RA. Therefore, based on these strong assumptions, this disease risk pattern could provide clinical researchers with multiple new research directions.

### Significant RA Risk Patterns of Associative Classification

A pattern contains multiple symptoms or diseases prior to a definite diagnosis of RA. These symptoms appear more often in the RA group, and rarely appear in the non-RA group. When a new patient’s diagnostic records contain these patterns, the patient has a higher risk of developing RA.

The RA early risk models were evaluated using ten-fold cross-validation, measured by sensitivity and specificity. The results are listed in Table 4. The average sensitivity was 80.3% and the average specificity was 72.6% across the ten experiments.

**Table 4:** Sensitivity and specificity of the RA disease risk model with ten-fold cross-validation.

Ten-fold	1	2	3	4	5	6	7	8	9	10
**Sensitivity**	78%	81%	78%	85%	82%	73%	84%	78%	82%	82%
**Specificity**	74%	72%	74%	73%	73%	71%	70%	73%	72%	74%

The disease risk patterns mined from the NHIRD can generally be categorized into two types. The relationship of Type I patterns is well known, as they are supported by ample research and are primarily single diseases associated with RA. The Type II patterns have no related research discussing their relationship with multiple diseases in a risk pattern.

#### Type I, well-known pattern

This type is primarily composed of a single disease or symptom that is typically known as an RA risk pattern and is covered in the literature. Examples of this pattern include {**rheumatism** ➔ **RA**, 82.97% confidence, 9.16% support, 4.9 relative risk}, {**autoimmune disease**, **not elsewhere classified** ➔ **RA**, 89.81% confidence, 7.02 relative risk}, {**arteritis, unspecified** ➔ **RA**, 72.58% confidence, 2.13% support, 2.64 relative risk} [22], {**systemic lupus erythematosus (SLE)** ➔ **RA**, 85.01% confidence, 3.6% support, 5.67 relative risk} (in the early stages of this disease, SLE patients may have joint complaints that are very similar to those observed in RA; this form of arthritis can be difficult to differentiate from RA) [23], and {**carpal tunnel syndrome (CTS)** ➔ **RA**, 73.95% confidence, 9.49% support, 2.8 relative risk} (CTS is one of the most frequent extra-articular manifestations of RA) [24]. The mining of Type I RA risk patterns is consistent with existing knowledge, and is supported by ample research.

#### Type II, unknown pattern

The majority of such patterns include multiple diseases or symptoms that combine to imply an RA risk pattern. Their joint relationship is unknown, and has rarely or never been discussed in the literature. These patterns are supported by high confidence, support, relative risk, and lift values, which are clinically valid hypotheses for the further study of their underlying medical significance. Related patterns include {**mitral valve disorders, chronic conjunctivitis, menopausal** ➔ **RA**, 83.57% confidence, 1.96% support, 5.1 relative risk}. In the literature, there is more discussion on the relationship between a single disease and RA; the relationship between multiple diseases and RA has been addressed far less often. Dismantling a portion of risk pattern mining from past studies may yield some relevant analysis, such as on mitral valve disorders and conjunctivitis.

Periodontal disease, an inflammatory problem of the tooth-supporting structures, may be an environmental trigger for RA [25]; indeed, it has been considered as a risk factor for RA. Evidence-based management of potential aggravating factors in subjects with active RA may be of clinical importance. Moreover, periodontal disease may be an aggravating factor for RA [26]. Laryngeal symptoms and laryngeal alterations are frequent in RA patients, which may indicate that RA and its treatment have laryngeal repercussions, even when the symptoms or morphological laryngeal involvement are not specific to RA [27]. Not all of these related diseases have been simultaneously discussed alongside RA in the literature; nevertheless, they may have been discussed with RA on an individual basis. Hidden relationships among multiple diseases within a risk pattern obviously exist. Similar phenomena were mined in the RA risk pattern set of the present study, such as {**laryngopharyngitis, periodontitis, rheumatism** ➔ **RA**, with a high confidence value of 85.29%, lift > 1 positive correlation, support of 2.45%, and high relative risk of 5.8}. This example shows that the disease risk patterns are consistent with previous literature; moreover, they can be used to make an assessment in the early disease phases, before a definite RA diagnosis has been made.

The relationship between single diseases and RA has frequently been discussed in the literature. For instance, many inflammatory disorders have been discussed alongside RA, such as asthma [28], atherosclerosis [29], conjunctivitis [30], cystitis [31], dental caries [32], enthesopathy [33], erosion of cervix disease, eczema, endocervicitis, fasciitis [34], gastroenteritis, laryngitis [27], Meniere’s disease, mitral valve disorders, carpal tunnel syndrome [24], periodontitis [35], pulpitis [36], synovitis and tenosynovitis [37], tonsillitis, ulcers [38], urticarial [39], and vaginitis [40]. However, the joint association of RA with diseases such as endocervicitis, gastroenteritis, pharyngitis, pulpitis, tonsillitis, urinary tract infections [41], and vaginitis has received less attention. Moreover, disease risk patterns composed of two to three diseases, which have the potential to form new hypotheses for clinical validation, are virtually unreported in the literature. The available information is summarized in Table 5.

**Table 5:** RA risk patterns of autoimmune-related diseases.

				Number of Related Papers	
RA Risk Patterns	Confidence	Support	RR	T1^a^	T2^b^
**Acute tonsillitis, unspecifiedautoimmune disease, myalgia and myositis**➔**RA**	93.09%	1.31%	13.5	20,1852,59,169	0
Benign neoplasm of breast, myalgia and myositis, **coronary atherosclerosis**➔**RA**	90.56%	1.64%	9.6	74,426,30	0
**Senile cataract**, unspecified **polyarthropathy**➔**RA**	90.46%	1.64%	9.5	4,25	0
**Adhesive capsulitis of shoulder**, **rheumatism**, joint pain ➔**RA**	90.29%	1.31%	9.3	15,1290,374	0
**Gastroenteritis and colitis**, **rheumatism**, irregular menstrual cycle➔**RA**	89.75	1.64%	8.75	16,5946,68	0
**Neurotic depression**, **osteoporosis**, cervicalgia (Neck pain)➔**RA**	88.74%	1.64%	7.9	916,1508,126	0
**Carpal tunnel syndrome**, osteoporosis, cervicalgia➔**RA**	88.49%	1.31%	7.7	123	0
**Unspecified inflammatory disease of female pelvic organs and tissues, rheumatism**➔**RA**	88.23%	1.8%	1.7	121	0
**Allergic rhinitis, synovitis and tenosynovitis, rheumatism➔RA**	88.06%	1.96%	7.4	62,2503,273	0
**Acute sinusitis, synovitis and tenosynovitis, rheumatism** ➔**RA**	87.45%	2.29%	6.9	49	0
**Eczema**, **asthma**, **urinary tract infections**➔**RA**	86.55%	1.64%	6.4	66,817,72	1
**Chronic hepatitis**, arthropathy, menopausal➔**RA**	84.94%	1.64%	5.6	794,662,137	0
**Mitral valve disorders**, **chronic conjunctivitis**, menopausal➔**RA**	83.57%	1.96%	5.1	41,25	0
**Meniere's disease**, myalgia and myositis, **contusion**➔**RA**	83.56%	1.64%	5.1	10,7	0
**Fasciitis, acute laryngitis**, arthropathy➔**RA**	77.14%	5.56%	3.4	47,5	0
**Synovitis** and **tenosynovitis**, arthropathy➔**RA**	76.58%	11.45%	3.2	21	2
**Acute upper respiratory infections**, arthropathy➔**RA**	74.80%	26.84%	2.9	744	2
**Dental caries**, **pulpitis**, arthropathy➔**RA**	72.95%	12.6%	2.7	13,2	0
**Acute cystitis**, arthropathy➔**RA**	72.9%	5.24%	2.7	42	0
**Periodontitis**, arthropathy➔**RA**	72.21%	16.36%	2.6	264	0
**Acute bronchitis**, arthropathy➔**RA**	70.75%	16.85%	2.4	65	1
**Acute laryngitis**, **myalgia and myositis**, **osteoarthritis**➔**RA**	70.64%	8.84%	2.4	5461	0
**Vaginitis and vulvovaginitis**, **acute upper respiratory infections**, joint pain➔**RA**	70.43%	8.35%	2.4	2,0	0
**Stomach disorder**, menopausal➔**RA**	70%	8.18%	2.3	122	0

Bold text denotes autoimmune-related diseases. The query term has been underscored, and repetitions are not marked again.

^a^T1 = Number of papers related to Type I pattern.

^b^T2 = Number of papers related to Type II pattern.

The experimental analysis of the NHIRD data indicates that a variety of autoimmune-related diseases could be considered RA assessment factors. This pattern is {(***unspecified disorder of joint OR rheumatism) AND autoimmune-related disease*** ➔ ***RA***} or {***multiple autoimmune-related disease*** ➔ ***RA***}, as shown in Table 5. Autoimmune symptoms that are minor in early RA progression may not be seriously considered; consequently, they may gradually develop into moderate and severe symptoms in different organs. However, if we can diagnose RA in early disease phases using RA disease risk patterns, then early treatment and control of the RA disease may be possible, improving patient quality of life and reducing the extraneous usage of medical resources.

Previous studies have rarely explored the causation of multiple simultaneous symptoms or diseases in patients with RA. Therefore, in the present study, we mined RA disease risk patterns from NHIRD. The disease risk patterns can each be supported by ample previous literature; however, they are rarely discussed together. Our results provide a strong topology for RA-related diseases. This topology enables population-based disease assessment prior to a definite diagnosis of RA, and thereby improves the clinical decision support available to physicians.

## Conclusions

In this paper, we have presented an effective framework for assessing RA premorbidity by discovering risk patterns in large-scale real data. The main contributions of this paper are as follows. First, we have illustrated the benefits of using a large-scale, nationally representative dataset, namely the Taiwanese NHIRD. Second, we integrated the associative classification technique with this dataset to derive an RA disease assessment framework for the analysis of RA disease risk. Our method is suitable and effective for large-scale population-based outpatient data, such as those in the NHIRD. The proposed framework assesses RA disease risk in patients before making a definite diagnosis of RA. This enables the discovery of risk patterns that allow for early diagnosis. Comprehensive experiments demonstrated the effectiveness of our framework, with an average sensitivity of 80.3% and an average specificity of 72.6%. In summary, the proposed framework facilitates the identification of many early risk patterns, including hidden risk factors, which are significantly associated with the target disease. Third, by summarizing disease risk patterns, we discovered a number of patterns that demonstrate a high correlation with RA prior to its diagnosis. In addition, we designed the Pattern Viewer, which provides physicians with further analysis of pattern characteristics by determining the comparative pattern distributions of RA and non-RA groups. The highest relative risk was found to occur in the psychiatric disorders category, and the highest proportion of RA risk patterns occurred in the musculoskeletal system category. These categories have higher relative ratios in the RA group. The risk ratios of discovered risk patterns were significant in the “240–279 endocrine, metabolic, and immunity,” “280–289 blood and blood-forming organs,” and “390–459 circulatory system” categories. Further, we developed an automated mechanism for searching the PubMed literature. This tool uses the NCBI e-utilities API to enable rapid confirmation of the degree of novelty and supporting knowledge of many disease risk patterns. Overall, our findings show that risk patterns mined in this study can be divided into two types, namely well-known patterns and unknown patterns. The well-known patterns are supported by previous literature, whereas the unknown patterns have rarely or never been discussed in past studies. This latter class has the potential to form a number of novel clinical research hypotheses.

In future work, when sequential risk pattern mining technique is used, it is very likely that more precise assessments could be provided by considering the development order of diseases. The proposed framework also has the versatility to be applied to the discovery of risk patterns in many major diseases by changing the target disease criteria in the patient grouping component.
